# Stimulation of Maize Growth and Development and Improvement of Soil Properties Using New Specialised Organic-Mineral Materials

**DOI:** 10.3390/molecules30143050

**Published:** 2025-07-21

**Authors:** Marzena S. Brodowska, Mirosław Wyszkowski, Ryszard Grzesik

**Affiliations:** 1 Department of Agricultural and Environmental Chemistry, University of Life Sciences in Lublin, Akademicka 15 Str., 20-950 Lublin, Poland; 2 Department of Agricultural and Environmental Chemistry, University of Warmia and Mazury in Olsztyn, Łódzki 4 Sq., 10-727 Olsztyn, Poland; 3 Jednostka Biznesowa Agro, Grupa Azoty Zakłady Azotowe Kędzierzyn S.A., Mostowa 30 A Str., 47-220 Kędzierzyn-Koźle, Poland; ryszard.grzesik@grupaazoty.com

**Keywords:** fertiliser materials, maize biomass, nutrients, soil

## Abstract

The use of mineral fertilisers has increased in recent years, but this has had a negative effect on the environment, including causing the water in rivers and lakes to become too rich in nutrients, a process known as eutrophication. Current research focuses on producing fertiliser materials that are environmentally friendly. The aim of this study was to examine the impact of novel specialised organic-mineral fertilisers (OMFs: NP 24-12, NP 10-10, and NP 10-10 Zn^+^) on the yield and chemical composition of maize. These fertilisers were compared with a control (no fertiliser) and with other fertilisers (mixture of commercial fertilisers (MCFs): NP 24-12 and NP 10-10) that were used as a reference. All fertilisers increased the SPAD index at the fifth leaf unfolded stage of maize, with the majority (apart from OMF NP 10-10) also increasing it at the panicle emergence stage. MCF NP 10-10 had the most positive effect on the plant height, while OMF NP 10-10 had the least positive effect. All fertilisers had a positive effect on maize growth and development, with MCFs NP 10-10 and NP 24-12 having by far the strongest effect on increasing crop yields. The yield of plants fertilised with OMFs NP 24-12, NP 10-10, and NP 10-10 Zn^+^ was lower than the yields of plants fertilised with MCF NP 24-12 and MCF NP 10-10. OMF NP 10-10 caused a greater increase in the contents of all elements, and OMF NP 24-12 caused a greater increase in most elements (except P and Ca) in maize than MCFs did at an identical NP ratio. OMF NP 10-10 Zn^+^ was found to have a significant impact on the mineral composition of maize, resulting in a decline in Ca and P levels, along with a notable increase in Mg, Zn, and Cu concentrations. The most significant differences were observed for Cu and Zn. The OMFs, notably NP 24-12 and NP 10-10, exhibited a comparatively diminished acidifying impact in comparison with the MCFs. The application of fertilisers resulted in a significant increase in soil nutrient levels, with most fertilisers increasing the soil N, P, and Zn contents. Additionally, the OMFs led to an increase in Cu. However, MCFs NP 24-12 and NP 10-10 reduced the soil Cu and Mn contents. Studies should include other species as they have different needs. Field experiments are also needed.

## 1. Introduction

As the world’s population grows, so does the demand for food and feed, both of which are essential for livestock farming. Therefore, producing high crop yields is vital to meet this demand. In order to produce high yields, plants need sufficient nutrients to support proper growth and development. However, the natural content in the soil environment is insufficient. These nutrients must therefore be supplied in the form of various environmentally safe fertilisers [1]. The excessive use of mineral fertilisers in recent years has led to excessive pollution of the environment, including the eutrophication of aquatic environments. The Nitrates Directive was enacted in the European Union to counteract this, stipulating that nitrogen fertilisers should only be used at certain times [2]. Concurrently, there has been a significant reduction in the use of mineral fertilisers, coupled with an increase in fertilisers containing organic components [3]. This is a highly beneficial phenomenon for the environment. However, it also forces fertiliser manufacturers to search for new materials that plants can absorb in large quantities without posing a threat to the environment, particularly to groundwater and, to a lesser extent, surface water. This is particularly challenging in the case of nitrogen fertilisers, as this nutrient is extremely poorly retained by the soil, especially when the fertilisers contain the nitrate ion [4]. Only the ammonium ion is partially bound in the soil sorption complex, extending its uptake time by plants [5].

The impact of fertilisation on maize yields is significantly influenced by climatic conditions, including the temperature, precipitation, and soil moisture, as well as the variability of these factors over time. Elevated temperatures can accelerate the mineralisation of organic fertilisers, thereby speeding up the release of nutrients. However, high temperatures can also cause heat stress, which impedes nutrient uptake by plants [6]. The distribution and quantity of precipitation are pivotal factors in determining the efficacy of fertiliser utilisation. Excessive rainfall can lead to nutrient leaching, while a lack of water can limit nutrient availability. It is important to recognise that optimal moisture conditions are essential for the efficient nutrient uptake by maize [7]. Elevated moisture levels have been shown to enhance fertiliser efficiency, while low moisture conditions have been shown to restrict nutrient availability. Seasonal climatic changes, including periods of drought or heavy rainfall, have been shown to affect the effectiveness of different types of fertiliser. Strachan and Jeschke [8] posited that the efficacy of organic fertilisers may be more effective under moderate conditions, whereas chemical fertilisers are more effective under extreme conditions.

Using mineral fertilisers alongside natural organic matter in soil produces the best results [9]. In practice, however, the use of organic fertilisers is only recommended for certain plant species. This does not mean that other plant species make poor use of nutrients from organic fertilisers. The reason for this is obvious: the production of organic fertilisers in developed countries is simply not high enough to meet the demand and apply them to all plant species [1]. Some plant species require fertiliser application at critical stages of growth. Mineral fertilisers are used for this purpose, and their components are rapidly taken up by the plants, preventing adverse effects resulting from their deficiency [10]. They are most often applied foliarly, and the time of their deactivation is relatively short, as part of the components taken up by the plants can be moved deep into the soil profile together with rainwater. Exclusive use of artificial fertilisers, particularly nitrogen and phosphorus fertilisers, can also reduce the soil microbial abundance [3].

Organic fertilisers have a beneficial effect on soil properties, including the sorption complex. They improve soil structure and porosity. This is the soil’s ability to retain water, which is particularly important during drought, as it extends the uptake of nutrients by plants [11,12]. Improved porosity and other properties are vital, as they make the soil more airy and able to retain water. Organic fertilisers also increase the number of soil microorganisms. These are essential for a healthy soil ecosystem, including the natural cycle of elements [12,13]. The slow mineralisation of organic fertilisers guarantees that plants have continuous access to nutrients [14]. This is vital, as it increases the utilisation rate of nutrients from fertilisers by plants, securing them in particular elements throughout the growing season [15]. However, it is important that the organic matter is properly prepared (processed) so that the nutrients can be released from it relatively quickly. The rate of mineralisation of organic manures depends on a number of factors, including the type of organic matter [15]. The process is fastest when the carbon-to-nitrogen ratio is low, the humidity and temperature are moderate (20–30 °C), the aeration is moderate, and the pH is close to neutral (about 6–7). It is also vital to ensure that the organic material is finely ground and has an adequate nitrogen content, as this is necessary for the microorganisms that decompose organic matter [16]. A study by Popin et al. [17] definitively showed that, of the several organic fertiliser materials, only by-products from the food industry performed better than mineral fertilisers in maize cultivation. Barquero et al. [14] stated that fertiliser materials containing at least some biological macronutrients are as effective as, if not more effective than, traditional mineral fertilisers in improving soil quality and plant growth.

It is important to note that different soil types have different capacities for retaining nutrients. Sandy soils have a reduced capacity to retaining water and nutrients, meaning fertilisers can be leached out of the soil more quickly. Conversely, clay soils have a superior capacity for retaining water and nutrients, thereby enhancing fertiliser efficiency. Mineral fertilisers resulted in significant yield increases in soils with a high humus content and better structure, while organic fertilisers were more beneficial in soils with low fertility and acidity. The use of organic fertilisers in sandy soil has been shown to improve water retention and soil structure, resulting in higher crop yields [18]. Using a combination of organic and inorganic fertilisers is key to improving both crop yields and soil quality. To understand the benefits of these two types of fertiliser, it is necessary to consider their effects on soil properties, the soil microbiome, nutrient availability, and plant physiological processes. Organic fertilisers offer the benefit of long-term improvements in soil structure and the soil microbiome, whereas inorganic fertilisers provide rapid access to nutrients. Taking into account the benefits of both types of fertiliser, proper fertiliser management can lead to sustainable yield increases and improved soil quality [15,19]. Clearly, alternative methods of supplying essential nutrients to plants must be found [20]. The use of organic-mineral fertilisers is essential [1]. These fertilisers combine the advantages of organic and mineral fertilisers by providing both organic substances and minerals. Consequently, they are highly beneficial for cultivating plants from which usable parts are extracted for food and fodder production [15]. They also reduce the depletion of natural resources and maintain soil productivity and fertility at an appropriate level, which is particularly positive for poorer soils [3,21]. Elbl et al. [1] definitively demonstrated that the use of various organic-mineral fertiliser materials significantly increased the contents of individual forms of mineral nitrogen (by over 10% on average) and humus (by more than 15%) in the soil. This resulted in an increase of more than 15% in the biomass yields of several crop species, including maize. It is also important to note that their effect on maize yield was greater than that of mineral fertilisers. The relationships described above triggered the development of new specialised organic-mineral fertiliser materials, which were used in the present study.

The aim of the study was to determine the effect of new specialised organic-mineral fertilisers (OMFs) on the yield and chemical composition of maize in terms of macronutrients (nitrogen, phosphorus, magnesium, and calcium) and micronutrients (zinc, copper, and manganese). The fertilisers applied were compared with a control (no fertiliser) and with reference fertilisers in the form of mixtures of commercial fertilisers (MCFs).

## 2. Results

### 2.1. Maize

#### 2.1.1. SPAD Index of Maize Leaves

**Figure 1:**
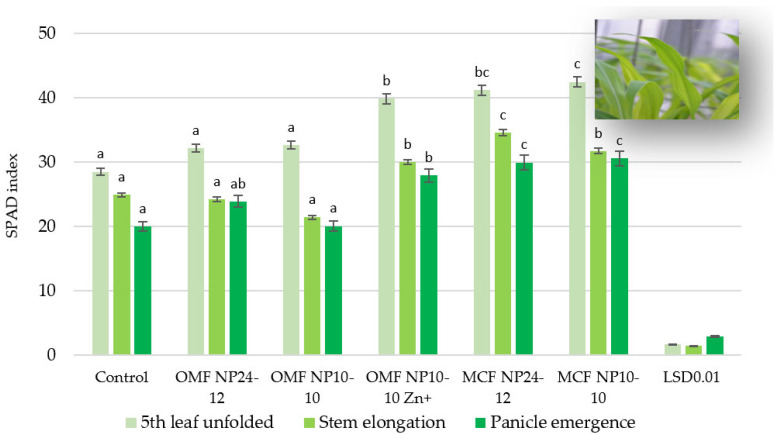
SPAD index of maize leaves in stages: fifth leaf unfolded, stem elongation, and panicle emergence. For each value, *n* = 5.

The SPAD index of maize leaves was highest at the fifth unfolded leaf stage and then decreased as the growing season progressed (Figure 1).

The SPAD index of maize leaves ranged from 28.49 to 42.50 at the fifth leaf unfolded stage, from 21.42 to 34.58 at the stem elongation stage and from 20.01 to 30.58 at the panicle emergence stage. At the fifth leaf unfolded maize stage, each of the fertilisers was applied to the soil, and at the panicle emergence stage, most of the fertilisers (with the exception of OMF NP 10-10) increased the SPAD index. At the stem elongation stage, their effect was less clear. Compared with the unfertilised object, the greatest increase in SPAD index values at the fifth leaf unfolded and panicle emergence stages was found after application of MCF NP 10-10 (by 49% and 53%, respectively), and the lowest increase was found after application of OMF NP 24-12 (by 13% and 19%, respectively). At the stem elongation stage, OMF NP 10-10 reduced the SPAD index value (by 14%), and OMF NP 10-10 Zn^+^, MCF NP 10-10, and MCF NP 24-12 increased the SPAD index value in maize leaves (by 20%, 27%, and 39%, respectively) compared with the unfertilised treatment. The SPAD index in maize leaves was lowered from 6% (fifth leaf unfolded) to 9% (panicle emergence) in the plants fertilised with OMF NP 10-10 Zn^+^, from 23% (fifth leaf unfolded) to 34% (panicle emergence) in the plants from pots with an application of OMF NP 10-10, and from 20% (panicle emergence) to 30% (stem elongation) after application of OMF NP 24-12 to the soil, compared with the MCFs with an identical NP content.

#### 2.1.2. Height of Maize

**Figure 2:**
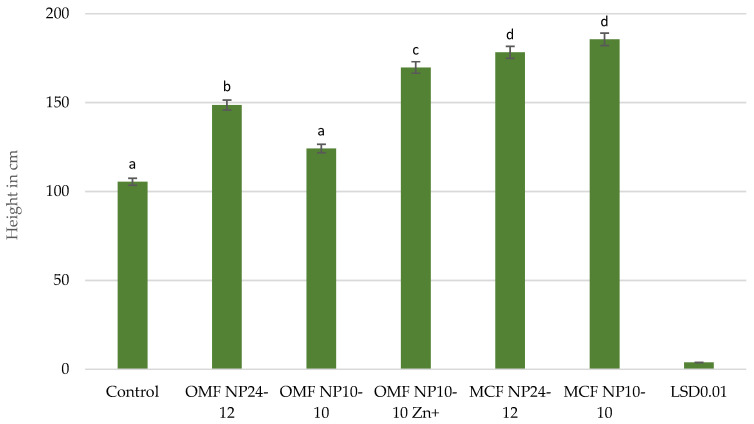
Height at panicle emergence stage (in cm) of maize. For each value, *n* = 5.

The plant height ranged from 105.5 cm to 185.6 cm (Figure 2). MCF NP 10-10 had the most beneficial effect on plant height, and OMF NP 10-10 had the least beneficial effect, causing increases in height of 76% and 18%, respectively, compared with the control. The maize height was 9%, 17%, and 33% lower at all sites treated with OMFs NP 10-10 Zn^+^, NP 24-12, and NP 10-10, respectively, compared with MCFs with an equivalent NP content.

#### 2.1.3. Yield of Maize

The yield of fresh weight of the above-ground parts of the maize ranged from 24.80 to 107.25 g per plant, and the yield of dry matter ranged from 3.61 to 15.48 g per plant (Table 1). The lowest yields of fresh and dry matter were found in the control. MCF NP 10-10 increased the fresh weight yield of the above-ground parts of maize by 332% and the dry matter yield by 328% compared with the unfertilised field. The yield=enhancing effect of MCF NP 24-12 on the yield of the above-ground parts of maize was lower, while that of OMF NP 10-10 was the lowest. Under their influence, there were increases of 268% and 135% in the fresh weight yield and 263% and 92% in the dry weight of the above-ground parts of maize, respectively, compared with the control. In contrast, the effect of OMF NP 10-10 Zn^+^ was stronger than those of OMF NP10-10 and OMF NP24-12. However, it should be noted that the effects of OMFs NP 24-12, NP 10-10, and NP 10-10 Zn^+^ were significantly lower than those of MCFs NP 24-12 and NP 10-10. The differences in yield of the maize’s above-ground parts were 14%, 46%, and 20% for the fresh weight and 28%, 55%, and 18% for dry matter, respectively.

#### 2.1.4. Macroelement Contents of Maize

The N content of the above-ground parts of the maize ranged from 7.09 g kg^−1^ of dry matter (DM) to 16.80 g kg^−1^ DM, while the P content ranged from 2.157 g kg^−1^ DM to 3.947 g kg^−1^ DM, the Mg content ranged from 1.538 g kg^−1^ DM to 2.459 g kg^−1^ DM, and the Ca content ranged from 3.033 g kg^−1^ DM to 4.263 g kg^−1^ DM (Table 1).

All fertilisers increased the N content in the above-ground parts of the maize. Compared with the unfertilised object, the increase in N in the above-ground parts of the maize ranged from 95% (OMF NP 10-10 Zn^+^ and MCF NP 24-12) to 137% (OMF NP 10-10), while the P content ranged from 41% (OMF NP 10-10 Zn^+^) to 83% (OMF NP 10-10), the Ca content ranged from 13% (OMF NP 10-10 Zn^+^) to 41% (OMF NP 10-10), and the Mg content ranged from 39% (MCF NP 10-10) to 60% (OMF NP 10-10 Zn^+^).

The N content of the above-ground parts of the maize treated with OMFs NP 24-12 and NP 10-10 were 13% and 18% higher, respectively, than the maize treated with MCFs with identical NP ratios. Similarly, the Ca and P contents of the maize increased by 10% and 12% (OMF NP 10-10), and the Mg content increased by 4% (OMF NP 24-12) and 15% (OMF NP 10-10 Zn^+^). However, OMF NP 10-10 Zn^+^ reduced the Ca and P contents in the above-ground parts of the maize by 11% and 13%, respectively, compared with the reference fertiliser.

#### 2.1.5. Microelement Contents of Maize

The content of Zn in the above-ground parts of the maize ranged from 32.77 mg kg^−1^ DM to 71.73 mg kg^−1^ DM, while the Cu content ranged from 1.867 mg kg^−1^ DM to 6.600 mg kg^−1^ DM, and the Mn content ranged from 48.00 mg kg^−1^ DM to 88.33 mg kg^−1^ DM (Table 1). The lowest levels of the analysed micronutrients were found in the control, while the highest levels were found in the series with OMF NP 10-10.

All fertilisers increased the content of the analysed micronutrients in the test plants. OMF NP 10-10 contributed most to this increase in the maize by up to 119% (Zn), 254% (Cu), and 84% (Mn) compared with the control. The MCFs had the least effect, namely NP 10-10 for Zn (+23%) and NP 24-12 for Cu (+34%) and Mn (+24%).

The contents of Zn and Cu in the above-ground parts of maize fertilised with all OMFs were higher than those of the MCFs with identical NP ratios, with the greatest effect found for Zn (+78%) by OMF NP 10-10 and for Cu (+128%) by OMF NP 24-12. OMFs NP 10-10 and NP 24-12 also increased the Mn content by 17% and 22%, respectively, compared with MCFs NP 10-10 and NP 24-12.

### 2.2. Soil Chemical Properties After Maize Harvest

#### 2.2.1. pH of Soil

The soil pH after maize harvesting ranged from 5.48 to 4.84 (Table 2). All fertilisers applied to the soil had an acidifying effect, especially the MCFs. The OMFs had a weaker acidifying effect than the MCFs, particularly NP 24-12 and NP 10-10.

#### 2.2.2. Macroelement Contents of Soil

The contents of the analysed macronutrients in the soil after maize harvesting were from 0.429 to 0.560 g kg^−1^ of dry matter (DM) for N, from 66.57 to 97.97 g kg^−1^ DM for P, from 0. 946 to 1.010 g kg^−1^ DM for Mg, and from 1.253 to 1.360 g kg^−1^ DM (Table 2) for Ca. Most of the fertilisers applied (with the exception of MCF NP 24-12) resulted in significant increases in the soil’s N content, ranging from 4% (OMF NP 24-12) to 15% (OMF NP 10-10 Zn^+^), and P concentration, ranging from 10% (OMF NP 24-12) to 47% (MCF NP 10-10), compared with the control. The N content was 4-7% higher in the OMF-fertilised soils than in the MCF-fertilised sites. On the other hand, the P content was 4–7% lower in the objects fertilised with OMFs NP 10-10 and NP 10-10 Zn^+^ and 8% higher in the soils fertilised with OMF NP 24-12. There was no significant effect from the applied fertiliser on the soil’s Mg and Ca contents.

#### 2.2.3. Microelement Contents of Soil

The contents of micronutrients in the soil after maize harvesting was similar in most of the studied objects and varied within the following ranges: from 17.55 to 24.22 mg kg^−1^ DM for Zn, from 1.593 to 3.987 mg kg^−1^ DM for Cu, and from 91.5 to 103.0 mg kg^−1^ DM for Mn (Table 2). The application of all fertilisers resulted in an increase in the soil Zn content ranging from 4% (MCF NP 10-10) to 38% (OMF NP 10-10 Zn^+^). The OMFs also increased the soil Cu concentration, ranging from 46% (NP 24-12) to 121% (NP 10-10 Zn^+^), compared with the control. In contrast, the MCFs reduced the Cu content by 7% (NP 24-12) and 12% (NP 10-10) and the Mn content by 10% and 8%, respectively. All OMFs had a more favourable effect on the Mn and Cu contents in particular, and OMFs NP 10-10 and NP 10-10 Zn^+^ also had a more favourable effect on the soil Zn concentration than the MCFs.

### 2.3. PCA Analysis

#### 2.3.1. Maize

**Figure 3:**
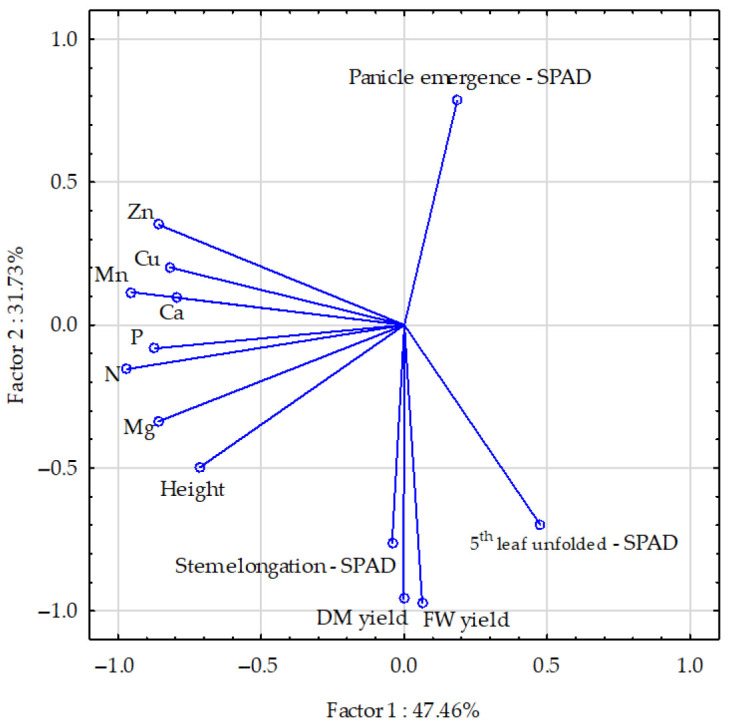
Maize yields and parameters, presented using the PCA method. The vectors represent the following parameters: fresh weight and dry matter yield; SPAD index in stages: 5th unfolded, stem elongation, and panicle emergence stages; height; and element (N, P, Mg, Ca, Zn, Cu, and Mn) contents in above-ground parts.

The majority of the vectors had similar lengths (with the exception of the SPAD index during the stem elongation stage), i.e., they were similarly important in explaining the proportion of variation (Figure 3). It was observed that only the vectors reflecting the N, Mo, and Fe contents were shorter than the others. Furthermore, negative correlations were identified between the SPAD index at panicle emergence and the maize’s fresh and dry matter yields, as well as the SPAD index at the stem elongation stage. Weaker correlations were observed at the fifth leaf unfolded stage of the plant. The contents of elements in the above-ground parts and the maize height were positively correlated. The strongest relationships were found between N and Mg and P; between Mg and plant height; and between Zn and Cu. Weaker relationships were found between Ca and Mn and P, between Cu and Mn, and between the SPAD index at the stem elongation stage and the maize yield.

#### 2.3.2. Soil After Maize Harvesting

**Figure 4:**
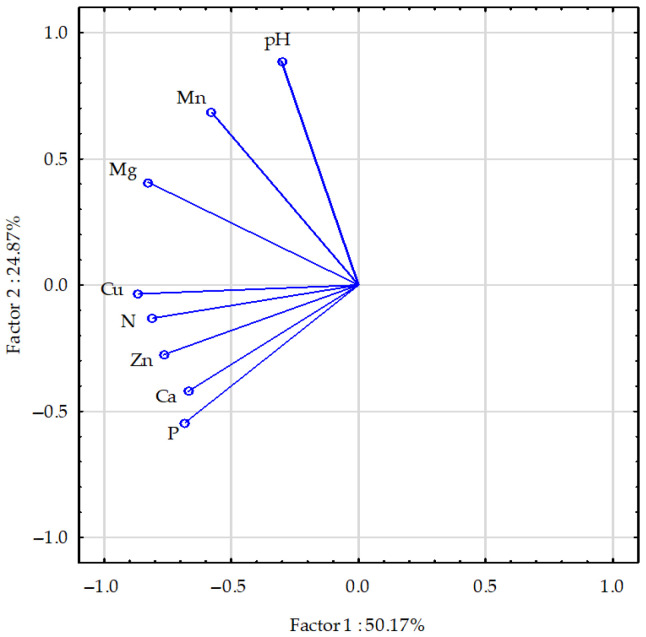
Selected maize post-harvest soil properties, presented using the PCA method. The vectors represent the following parameters: pH and element (N, P, Mg, Ca, Zn, Cu, and Mn) contents.

Most of the vectors representing soil properties were similar in length. Only the vectors representing the Ca and Mg contents were slightly longer (Figure 4).

The correlations between the contents of the individual elements were positive, with particularly strong correlations between Ca and P and Zn as well as between N and Zn and Cu. Weaker correlations were identified between P and Zn; between Mg and Cu and Mn; between Cu and Zn; and between the pH and Mn in the soil. A relatively weak negative correlation was also identified between the soil pH and the contents of P, Ca, and Zn.

## 3. Discussion

In order to achieve high yields of plants of adequate quality, it is essential to provide them with fertilisers that meet their nutritional needs throughout the growing season. This is particularly important during critical phases when plants absorb large quantities of nutrients [22]. Organic-mineral fertilisers seem to fulfil these conditions due to their multiple effects on the plant properties, growth and development, and chemical composition [15,23]. According to Barquero et al. [14], fertiliser materials of biological origin are less effective but perform identically to or even better than conventional mineral fertilisers by the end. Materials of biological origin also increase the abundance of soil microorganisms. The soil microorganisms are particularly important in determining the availability of mineral nutrients to plants. They therefore determine soil fertility [24]. Organic-mineral fertilisers are most commonly used in horticulture and much less commonly in typical crop production. This highlights the urgent need for research in this area.

This research suggests that such fertilisers have considerable potential for use in producing silage maize. Among the fertilisers tested in the trial, OMF NP 24-12 and OMF NP 10-10 Zn^+^ had the most favourable effect on the maize yield; however, their effect was smaller than that of the MCFs with the same NP ratio. It should also be noted that OMF NP 10-10 (despite its lower effect on the maize yield) also had a positive effect on the plants, causing a greater increase in the contents of all elements (except P and Ca) in maize than MCFs.

The effect of fertilisers on the yields and biometric characteristics of plants was primarily due to their basic macronutrient contents, i.e., N and P (as these fertilisers were produced using N-P fertilisers). However, the micronutrient contents also played an important role in the growth and development and, consequently, the yields of the above-ground parts of maize. The relationship between the SPAD index, which indicates leaf greenness and chlorophyll content, and maize yield and biometric traits is well documented in the scientific literature [25,26]. The SPAD index is a measure of leaf greenness that correlates with the chlorophyll content. Chlorophyll is imperative for photosynthesis, and its presence affects the efficiency with which sunlight is converted into chemical energy [25]. Higher SPAD values, which indicate a greater chlorophyll content, often correlate with higher maize yields. Clearly, chlorophyll significantly influences the capacity of plants to engage in photosynthesis, a process that is intricately linked to the biomass generation and the subsequent yield. The SPAD index has been demonstrated to be associated with various biometric maize traits, such as plant height, leaf number, and grain weight [26]. Nitrogen fertilisers (especially urea and ammonium nitrate) increase the SPAD value during the early stages of maize growth, especially during the stem elongation stage, consequently having a favourable effect on the plants’ yields [27]. Organic materials, especially those rich in humic substances, have an analogous effect; however, their impact on the SPAD index, plant height, and yield is generally smaller than that of mineral fertilisers, although positive [27]. In a study by de Morais et al. [9], applying OMFs, especially on poor soils, also increased the maize biomass. In Wapa’s experiment [28], combining nitrogen fertiliser with organic matter induced the greatest increase in maize yield and positively affected its parameters (plant height, number and area of leaves per plant, and number of grains per cob). The positive effect of organic-mineral fertilisers on crop yields has also been demonstrated by Romanowska-Duda et al. [29]. El-Habbak et al. [30] also demonstrated their positive impact on plant biometric characteristics, resulting in increased yields. Increasing the organic matter content of OMFs can improve the efficiency of maize nutrition. Under its influence, maize biomass increases more than it does after the application of mineral fertiliser alone, especially in poor soil conditions; experimental studies indicated increases in maize shoot biomass yields of 26% and 22%, respectively. In contrast, the effect on the maize yield was slightly lower but still positive in organic-rich soils [16]. In a study by Elbl et al. [1], the addition of OMF resulted in a slightly smaller increase in the maize biomass yield (over 15%), but the effect was greater than that of mineral fertilisers alone. In an experiment by El-Habbak et al. [30], the combination of organic materials and mineral fertilisers increased plant yields by 56%, outperforming mineral fertilisers alone. According to Boscaro et al. [31], the increase in maize biomass yields due to OMFs can even greater, reaching up to 80% in their experiments. Organic-mineral fertilisers increased the root biomass (by up to 80%) and root length (by more than 60%), promoting more intensive nutrient uptake from the soil by maize and contributing to increased crop yields [31].

Maize is one of the world’s most important crops. To produce high yields, it requires large amounts of nitrogen and other elements [17]. When it comes to nitrogenous fertilisers, the way in which N is supplied to the plant—either through the roots or leaves—is important. Plants take up nitrogen from the soil in the form of NH_4_^+^ and NO_3_^−^ ions, with NO_3_^−^ ions being more mobile. This is advantageous in the case of fertilisation intervention. This is mostly positive, as NO_3_^−^ ions are more easily leached into the soil than NH_4_^+^ ions, which are partly partially absorbed by the soil. Therefore, it is optimal to provide plants with both forms of N throughout the growing season [5]. This is especially important if the fertiliser contains macronutrients and micronutrients that are essential for plant growth and development. The fertilisers used in our experiment met these criteria, containing not only N and P but also several essential micronutrients, which were then tested in the soil and plants. OMFs have a positive effect on the mineral N content of soils [1] and on P, K, Mg and Ca, especially in lighter soils [32]. The application of organic matter as a fertiliser has a positive effect on the soil pH [33] and organic C [33], N [21,33], P [33,34], K [33,34], Mg [34], and Ca [34], while the application of mineral fertiliser alone can lead to a reduction in their soil values after harvesting [33]. In an experiment by Elbl et al. [24], an increase of over 10% in the soil N content was observed following the application of an OMF, which also had a positive impact on the soil organic matter content. Jaskulska et al. [35] demonstrated that OMF increased the soil pH as well as the K and Mg contents. Our study clearly showed that there were increases in the contents of all macronutrients and micronutrients in maize as well as increases in the soil levels of N, K, Cu, and Zn after harvesting. It should also be emphasised that among the tested OMFs, OMF NP 10-10 had the greatest effect on plant accumulation, while OMF NP 24-10 Zn^+^ had the greatest effect on the soil N, P, Mg, Cu, and Zn contents. In poor soils, the Fe and Mn contents of maize can increase after the application of OMFs, as this changes the dynamics and improves the utilisation efficiency of these elements [9]. Accordingly, the contents of trace elements are incredibly important during plant growth and development as these elements are involved in many physiological processes, including the activation of numerous enzymatic reactions [36].

According to de Morais et al. [9], higher levels of organic matter content in OMF can reduce the Mn content in the soil solution. However, soils with higher organic matter contents show an increase in their Fe and Cu contents, as well as Zn availability in soils with higher organic matter contents. Conversely, a decrease in Fe content occurs as the amount of organic matter in the soil decreases. An increase in trace elements in soil has led to an increase in their accumulation in plants. This has a beneficial effect on their growth and development [37,38]. However, according to Chen et al. [39] and Wyszkowski et al. [32], applying organic matter to soil can reduce the availability of certain trace elements to plants. This is particularly important in soils contaminated with heavy metals. Applying organic matter in combination with classical mineral fertilisers contributes to the neutralisation of acidity in the soil solution. On the other hand, the application of mineral fertilisers alone has no such effect and can increase soil acidity, as clearly shown by Litvinova and Dehodiuk [40].

The effect of organic-mineral fertilisers on soil properties is particularly important due to their positive impact on soil biology, including increasing the abundance of soil microorganisms involved in the elemental cycle [15,24]. An increase in soil organic carbon, which serves as an energy source for these microorganisms, is mainly associated with an increase in their abundance following the application of organic-mineral fertilisers. This is particularly important in the later stages of vegetation, when soil microorganisms positively affect plant nutrient availability [14]. This increased nutrient availability leads to intensive growth of plant biomass and the development of its generative parts, which is particularly important for cereal crops. The result is an increase in plant yield, which is characterised by a favourable chemical composition, particularly with regard to the contents of macronutrients and trace elements. These nutrients are available to the plant for a longer period of time than traditional mineral fertilisers due to the gradual release of elements from the mass of fertiliser material [15].

The presence of zinc in organic-mineral fertilisers (OMFs) increases maize’s uptake of essential nutrients necessary for growth and development, such as N, P, and K. This is due to Zn’s role in enzyme activity and root development, which increases nutrient uptake by plants [41]. Zinc is involved in the synthesis of auxins, the primary growth hormones responsible for root and above-ground growth [42]. Enriching OMFs with Zn enhances the defence mechanisms of plants against diseases and environmental stresses, thus improving plant health. Zinc is also involved in protein formation and plays an important role in regulating oxidative stress, which is particularly important under stressful conditions such as water deficiency, acidification, and salinity [43]. Using OMFs containing Zn ensures a balanced supply of the various elements necessary for plant growth and development by affecting nutrient availability and uptake, which contributes to higher yields and better crop quality [41]. The nutrient contents of plants is then correlated with their presence in the soil, especially in the root zone, and the application of OMFs (including those with Zn) improves soil fertility by stimulating microbial activity [42] and increasing the availability of essential elements in the soil as a result of mineralisation of organic parts [43], leading to improved soil structure, health, and fertility [42].

The positive effect of organic-mineral fertilisers on maize and other plants is mainly due to their beneficial impact on the slow release of nutrients, which coincides with the different phases of plant growth [44]. For example, Elrys et al. [45] found that the long-term application of OMFs in combination with synthetic nitrogen fertilisers increased the mineralisation rates of both organic and labile organic N, as well as the release of adsorbed ammonium from cation exchange sites. In contrast, synthetic fertilisers alone only increased the mineralisation rate of organic N. Therefore, nutrient losses from soil due to N release, P fixation, or K leaching are lower than when classical mineral fertilisers are used [46]. OMFs also have a beneficial effect on the growth of soil microorganisms [47], accelerating the decomposition of organic compounds and increasing the availability of nutrients to plants. This results in increased biomass and higher levels of individual elements in plants, positively affecting their quality and the formation of both vegetative and generative parts [48].

Studies have shown that using organic-mineral fertilisers, rather than separate mineral and organic fertilisers, is clearly beneficial to plants and the soil environment. However, these studies should be extended to include other plant species, as they have different nutrient and fertiliser requirements. Field experiments should also be conducted.

## 4. Materials and Methods

### 4.1. Methodological Assumptions

The one-year vegetative pot experiment was conducted in the vegetation hall of the University of Warmia and Mazury in Olsztyn, Poland (latitude 53.78° N, longitude 20.49° E). The soil material used in the pot experiments was obtained from the arable layer of typical brown soil (Eutric Cambisol) located at the Tomaszkowo Teaching and Experimental Centre (latitude 53.72° N, longitude 20.42° E, Poland), which belongs to the University of Warmia and Mazury in Olsztyn, Poland. According to the soil classification, the soil was categorised as follows: the sands group and loamy sands subgroup [49]. The soil’s granulometric composition was as follows: a sand fraction (2.0–0.05 mm) of 91.88%, dust fraction (0.05–0.002 mm) of 7.44%, and clay fraction (below 0.002 mm) of 0.68%. The soil’s characteristics were as follows: pH = 6.33, nitrogen = 0.747 g N kg^−1^ dry matter (DM), phosphorus = 77.77 mg kg^−1^ DM, magnesium = 0.869 g kg^−1^ DM, calcium = 0.840 g kg^−1^ DM, zinc = 28.99 mg kg^−1^ DM, copper = 3.567 mg kg^−1^ DM, and manganese = 136.5 mg kg^−1^ DM.

The experiment comprised a series of pot experiments to investigate the effect of applying three different, new specialised solid organic-mineral fertilisers (OMFs) (NP 24-12, NP 10-10, and NP 10-10 Zn^+^) to soil. These were compared with a control (no fertiliser) and two other complex fertilisers (mixture of commercial fertilisers (MCFs): NP 24-12 and NP 10-10). The experiment was conducted to assess the impact of soil application of these fertilisers, in combination with a control (no fertiliser), on the yield of the above-ground parts and chemical composition (N, P, Ca, Mg, Zn, Cu, and Mn) of maize for foraging under abiotic stress conditions (abiotic drought and reduced light). New specialised organic-mineral fertilisers (OMFs) were developed at Grupa Azoty Zakłady Azotowe Kędzierzyn S.A. (Kędzierzyn-Koźle, Poland) as part of the project “New formulations of specialised organo-mineral fertilisers” (PO-IR.01.02.00-00-0029/17). These fertilisers were produced through the large-scale batch granulation of mineral components (ammonium nitrate, urea, and ammonium phosphates) and organic components (waste from the food industry and algae). The algae-waste mixture content in the produced fertilisers was approximately 24–25% by weight. The exact composition of the mixture varies depending on the specific fertiliser formulation. The fertilisers were also enriched with microelements through the addition of inorganic salts. The micronutrient contents of the fertiliser formulations were as follows: Zn = 0.17% (NP 24-12), 0.37% (NP 10-10), and 0.56% (10-10 Zn^+^); Cu = 0.17% (NP 24-12), 0.15% (NP 10-10), and 0.19% (10-10 Zn^+^); and Mn = 0.25% (NP 24-12), 0.36% (NP 10-10), and 0.36% (10-10 Zn^+^). Due to the phenomenon of potential decomposition of ammonium nitrate, the nitrogen content was limited to 10% by weight. Fertilisers with a higher nitrogen content contain urea in their composition. The above fertilisers are in the process of being patented. The test crop was maize of the Polish variety Lokata, and the trials were performed in 5 replications. Maize was chosen for the study due to its significant role in the global food and feed economy.

**Figure 5:**
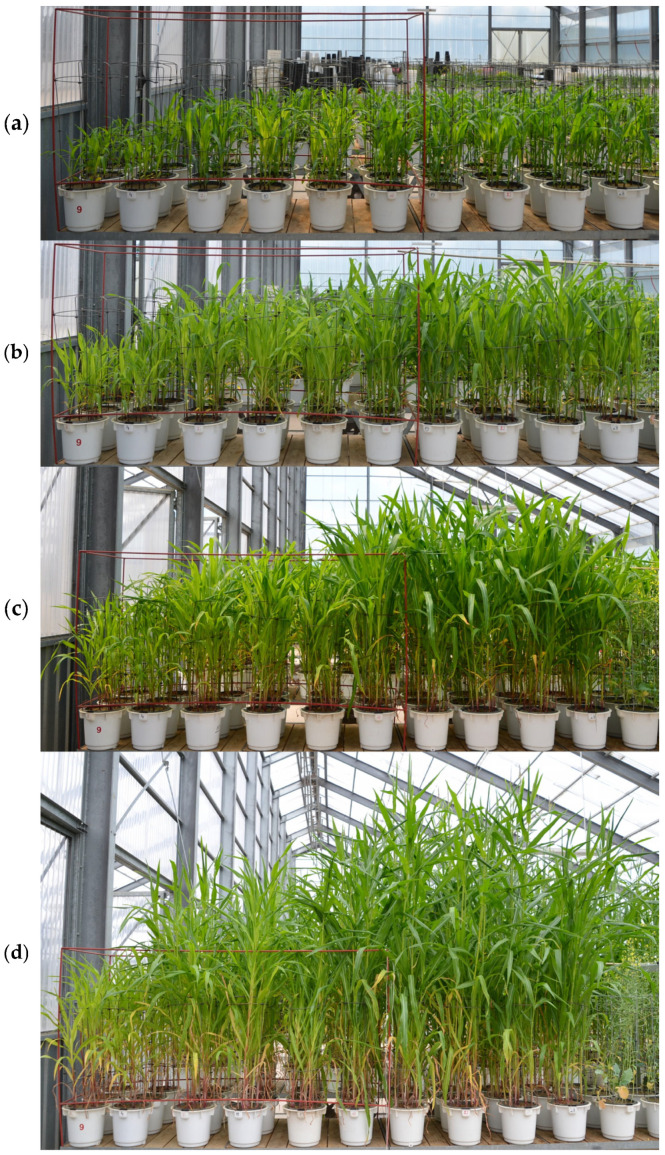
Maize at different vegetative stages: (**a**) leaf development (BBCH 15), (**b**) 9 leaves (BBCH 19), (**c**) stem elongation (BBCH 39), and (**d**) panicle development (BBCH 59).

Prior to planting the test plants, the pots were fertilised with potassium at doses adapted to the nutritional requirements of the test plants (140 mg N kg^−1^ and 100 mg K_2_O kg^−1^ of soil). Phosphorus doses were either half of the nitrogen dose (OMF NP 24-12 and MCF NP 24-12) or the same as the nitrogen dose (OMF NP 10-10, OMF NP 10-10 Zn^+^, and MCF NP 10-10). The solid organic-mineral fertilisers were applied to the soil in accordance with the recommendations for conducting pot experiments with maize. The OMFs and MCFs were applied at rates of 0.583 g per kg of soil (for NP 10-10) and 1.400 g per kg of soil (for NP 24-12), respectively. The fertilisers were thoroughly mixed into the soil (9 kg per pot) when the experiment was set up. Maize seeds were then sown on 4 May at a target density of 8 plants per pot. During the plants’ growing period, the soil moisture in the pots was maintained at a constant level corresponding to the stress conditions (50% of the field water capacity). The soil in the pots was watered with distilled water to eliminate the possible, although relatively small, influence of elements found in natural tap water. Agro textile was used periodically to shade the experiment and observe how the plants would respond to light stress. The vegetation hall was not temperature-regulated. The average monthly air temperature ranged from 12.1 °C in May to 17.9 °C in June and 18.0 °C in July. The humidity ranged from 66% in May to 74% in June and 73% in July. The total precipitation ranged from 35.5 mm in May to 92.5 mm in June and 55.9 mm in July. The insolation was 250.9 h in May, 284.7 h in June, and 269.9 h in July. The atmospheric pressure was 1000.2 hPa in May, 999.9 hPa in June, and 1000.8 hPa in July. On days without rain, fresh air was supplied to the vegetation hall through open windows. Its transparent glass roof and side walls allowed natural lighting of the plants, in keeping with the natural conditions outside. The experiment was shaded by covering it with agro textile for a period of two days per week. The characteristic developmental stages of the maize were photographed during the course of the study (Figure 5).

The first maize panicles were found in pots that had been fertilised with the NP 10-10 commercial fertiliser mixture. The crop was harvested at the panicle emergence stage (BBCH 59). After harvesting of the plants, the following parameters were determined: the yields of fresh and dry matter of the above-ground parts of the maize. Additionally, during vegetation, the leaf greenness index (SPAD) was measured during vegetation at the following stages: 5th leaf unfolded (BBCH 15 on 23 May), stem elongation (BBCH 31 on 20 June), and panicle emergence (BBCH 59 on 12 July). At the point of harvest (12 July), the plant height was measured, and plant and soil samples were collected for subsequent laboratory analysis. At harvest (12 July), the plant height was measured, and the plants and soil samples were collected for subsequent laboratory analysis.

### 4.2. Laboratory and Statistical Analysis Methods

In this study, the elemental composition (N, P, Ca, Mg, Zn, Cu, and Mn) of plant material (above-ground parts of maize) was analysed in new specialised OMFs. The study investigated the potential impact of these fertilisers on the uptake and accumulation of elements in plants. Soil samples were also analysed for their elemental contents and granulometric compositions.

The greenness of the maize leaves (Soil and Plan Analysis Development (SPAD) index) was measured using a Konica Minolta Optics SPAD-502Plus meter (Konica Minolta, Inc., Chiyoda, Tokyo, Japan). The plant samples were crushed, dried, and ground. Soil samples were air-dried and sieved through a 2 mm mesh sieve. Laboratory analyses of the plants and soil were performed using the following methods: Kjeldahl distillation (N) [50], calorimetric vanadium-molybdenum (P) [51], and atomic absorption spectrometry (AAS) (Ca, Mg, Zn, Cu, and Mn) [51]. H_2_SO_4_ was used to digest plant and soil samples in a Speed-Digester K-439 for macronutrient content, and HNO_3_ (for plants) and royal water (for soil) were used in a MARS 6 microwave digestion system (CEM Corporation, Matthews, NC, USA) for the micronutrients. To ensure the accuracy of the laboratory analysis results, standard reference materials from Fluka (Charlotte, NC, US) as well as NCS ZC 73030 CRM and Soil S-1 CRM were used. The pH in 1 M KCl was determined potentiometrically [52], and the granulometric composition was determined using the laser diffraction method [53].

Statistical analysis was conducted using the STATISTICA 13.3 software package [54], with one-way analysis of variance (ANOVA) and Tukey’s honestly significant difference (HSD) test (*p* ≤ 0.01) in conjunction with principal component analysis (PCA). This approach enabled the estimation of correlations between the SPAD index, plant height and yield, and element contents in plants and soil, as well as the soil pH.

## 5. Conclusions

The SPAD values, which indicate greenness, were highest at the fifth leaf unfolded stage and decreased as the growing season progressed. All fertilisers increased the SPAD index at the fifth leaf unfolded stage of the maize, and most of them (with the exception of OMF NP 10-10) also increased it at the panicle emergence stage. MCF NP 10-10 had the most positive effect on the plant height, whereas OMF NP 10-10 had the least positive effect. All fertilisers used had a positive effect on maize growth and development, with MCF NP 10-10 and MCF NP 24-12 having by far the strongest yield-enhancing effect. The yields of plants fertilised with OMFs were as follows. The yields for NP 24-12, NP 10-10, and NP 10-10 Zn^+^ were 14, 46, and 20% lower (fresh weight) and 28, 55, and 18% lower (dry weight), respectively, than those of the plants fertilised with MCF NP 24-12 and MCF NP 10-10, respectively. OMF NP 10-10 caused a greater increase in the contents of all elements, and OMF NP 24-12 caused a greater increase in most elements (except P and Ca) in maize than MCFs did at an identical NP ratio. OMF NP 10-10 Zn^+^ contributed to a decrease in the Ca and P contents as well as an increase in the Mg, Zn, and Cu concentrations in maize. The greatest differences were observed for Cu and Zn.

The OMFs, especially NP 24-12 and NP 10-10, had a weaker acidifying effect than the MCFs. All or most of the fertilisers applied increased the soil N, P, and Zn contents, and the OMFs also increased the Cu content. In contrast, MCFs NP 24-12 and NP 10-10 reduced the soil Cu and Mn contents.

Nevertheless, the present study should be extended to include other plant species, as these possess different nutrient and fertiliser requirements. The execution of field experiments is also recommended.

## Figures and Tables

**Table 1:** Impact of new specialised organic-mineral fertilisers on maize.

Fertiliser	Yield FM	Yield DM	N	P	Mg	Ca	Zn	Cu	Mn
	g Plant^−1^			Content in g kg^−1^ DM			Content in mg kg^−1^ DM		
Control	24.80 ± 0.36	3.61 ± 0.24	7.09 ± 0.12	2.157 ± 0.065	1.538 ± 0.128	3.033 ± 0.129	32.77 ± 1.29	1.867 ± 0.115	48.00 ± 3.00
OMF NP24-12	78.38 ± 0.60	9.41 ± 0.10	15.59 ± 0.27	3.077 ± 0.180	2.341 ± 0.111	3.953 ± 0.414	54.73 ± 5.00	5.700 ± 0.693	72.33 ± 5.39
OMF NP10-10	58.38 ± 1.01	6.95 ± 0.40	16.80 ± 0.12	3.947 ± 0.100	2.403 ± 0.048	4.263 ± 0.283	71.73 ± 4.38	6.600 ± 0.200	88.33 ± 5.03
OMF NP10-10 Zn^+^	85.35 ± 0.98	12.71 ± 0.31	13.81 ± 0.43	3.050 ± 0.095	2.459 ± 0.062	3.430 ± 0.156	49.95 ± 2.65	6.133 ± 0.513	74.33 ± 6.43
MCF NP24-12	91.28 ± 1.13	13.13 ± 0.39	13.81 ± 0.43	3.070 ± 0.166	2.243 ± 0.277	3.893 ± 0.380	45.97 ± 0.81	2.500 ± 0.173	59.33 ± 6.43
MCF NP10-10	107.25 ± 1.50	15.48 ± 0.34	14.28 ± 0.36	3.523 ± 0.220	2.143 ± 0.137	3.863 ± 0.327	40.23 ± 5.36	3.533 ± 0.231	75.67 ± 4.16
LSD_0.01_	0.19	0.14	0.79	0.626	0.369	0.752	7.52	0.957	13.18

OMF = organic-mineral fertiliser, MCF = mixture of commercial fertilisers, DM = dry matter. Average values ± standard deviation. For each value, *n* = 5.

**Table 2:** Impact of new specialised organic-mineral fertilisers on soil properties after harvesting of maize.

Fertiliser	pH	N	P	Mg	Ca	Zn	Cu	Mn
			Content in g kg^−1^ DM			Content in mg kg^−1^ DM		
Control	5.48 ± 0.02	0.485 ± 0.032	66.57 ± 2.26	0.989 ± 0.015	1.253 ± 0.023	17.55 ± 1.09	1.807 ± 0.147	101.7 ± 1.1
OMF NP24-12	5.20 ± 0.06	0.504 ± 0.010	73.33 ± 2.22	0.962 ± 0.017	1.287 ± 0.012	18.74 ± 0.28	2.640 ± 0.072	100.7 ± 3.3
OMF NP10-10	5.18 ± 0.03	0.523 ± 0.032	94.40 ± 2.19	0.998 ± 0.022	1.360 ± 0.040	21.95 ± 1.43	3.147 ± 0.196	103.0 ± 2.1
OMF NP10-10 Zn^+^	5.02 ± 0.05	0.560 ± 0.005	91.44 ± 1.83	1.010 ± 0.014	1.313 ± 0.012	24.22 ± 0.55	3.987 ± 0.172	98.9 ± 3.3
MCF NP24-12	4.94 ± 0.03	0.429 ± 0.033	68.03 ± 2.49	0.946 ± 0.013	1.260 ± 0.035	19.17 ± 0.59	1.680 ± 0.072	91.5 ± 1.2
MCF NP10-10	4.84 ± 0.04	0.523 ± 0.032	97.97 ± 2.47	0.970 ± 0.014	1.313 ± 0.061	18.29 ± 0.48	1.593 ± 0.050	93.4 ± 1.7
LSD_0.01_	0.23	0.067	5.63	0.041	0.087	2.09	0.327	5.7

OMF = organic-mineral fertiliser, MCF = mixture of commercial fertilisers, DM = dry matter. Average values ± standard deviation. For each value, *n* = 5.

## Data Availability

Data are contained within the article.
